# Crosstalk Between Metabolism and Immune Activity Reveals Four Subtypes With Therapeutic Implications in Clear Cell Renal Cell Carcinoma

**DOI:** 10.3389/fimmu.2022.861328

**Published:** 2022-04-11

**Authors:** Yi Wang, Xin-De Zheng, Gui-Qi Zhu, Na Li, Chang-Wu Zhou, Chun Yang, Meng-Su Zeng

**Affiliations:** ^1^ Department of Radiology, Zhongshan Hospital, Fudan University, Shanghai Institute of Medical Imaging, Shanghai, China; ^2^ Department of Cancer Center, Zhongshan Hospital, Fudan University, Shanghai, China; ^3^ Department of Liver Surgery and Transplantation, Liver Cancer Institute, Zhongshan Hospital, Fudan University, Shanghai, China

**Keywords:** metabolism, immunotherapy, immune activity, radiomic analysis, muti-omics analysis

## Abstract

Clear cell renal cell carcinoma (ccRCC) is characterized by metabolic dysregulation and distinct immunological signatures. The interplay between metabolic and immune processes in the tumor microenvironment (TME) causes the complexity and heterogeneity of immunotherapy responses observed during ccRCC treatment. Herein, we initially identified two distinct metabolic subtypes (C1 and C2 subtypes) and immune subtypes (I1 and I2 subtypes) based on the occurrence of differentially expressed metabolism-related prognostic genes and immune-related components. Notably, we observed that immune regulators with upregulated expression actively participated in multiple metabolic pathways. Therefore, we further delineated four immunometabolism-based ccRCC subtypes (M1, M2, M3, and M4 subtypes) according to the results of the above classification. Generally, we found that high metabolic activity could suppress immune infiltration. Immunometabolism subtype classification was associated with immunotherapy response, with patients possessing the immune-inflamed, metabolic-desert subtype (M3 subtype) that benefits the most from immunotherapy. Moreover, differences in the shifts in the immunometabolism subtype after immunotherapy were observed in the responder and non-responder groups, with patients from the responder group transferring to subtypes with immune-inflamed characteristics and less active metabolic activity (M3 or M4 subtype). Immunometabolism subtypes could also serve as biomarkers for predicting immunotherapy response. To decipher the genomic and epigenomic features of the four subtypes, we analyzed multiomics data, including miRNA expression, DNA methylation status, copy number variations occurrence, and somatic mutation profiles. Patients with the M2 subtype possessed the highest VHL gene mutation rates and were more likely to be sensitive to sunitinib therapy. Moreover, we developed non-invasive radiomic models to reveal the status of immune activity and metabolism. In addition, we constructed a radiomic prognostic score (PRS) for predicting ccRCC survival based on the seven radiomic features. PRS was further demonstrated to be closely linked to immunometabolism subtype classification, immune score, and tumor mutation burden. The prognostic value of the PRS and the association of the PRS with immune activity and metabolism were validated in our cohort. Overall, our study established four immunometabolism subtypes, thereby revealing the crosstalk between immune and metabolic activities and providing new insights into personal therapy selection.

## Introduction

Metabolic reprogramming and immune infiltration, which are two hallmarks of cancer progression, interact with each other in diverse ways in the tumor milieu (1–3). Prior studies confirmed that tumor cells tended to outcompete T cells for glucose, which would restrict the uptake of glucose in effector T cells, thereby dampening their tumor clearance abilities (4, 5). However, regulatory T cells have a distinct metabolic profile in the TME and are involved in alternative metabolic pathways, such as the lipid signaling pathway, to stabilize the supply of cellular energy under glucose deficiency conditions (6). In addition, a high rate of cholesterol esterification in the tumor was demonstrated to compromise T-cell responses (7, 8). The above evidence indicated that the perturbation of metabolism could influence the status of immune cells and vice versa. Therefore, considering the heterogeneity and complexity of the TME, combining metabolic therapy with immunotherapy could enhance therapeutic efficacy. A recent study pointed out that glycolysis-low tumors were more responsive to CTLA-4 blockade, suggesting that weakening metabolic competition could improve the effectiveness of immunotherapy (9).

Clear cell renal cell carcinoma (ccRCC), the most prevalent subtype of renal cell carcinoma, is considered an immunogenic disease (10, 11). Accumulating evidence shows that ccRCC is normally in an immunosuppressive state, which is characterized by the high infiltration of regulatory T cells and myeloid cells with suppressive activity (12, 13). Although immune checkpoint inhibition (ICI) therapy is supposed to be effective in restoring the antitumor immune response for ccRCC patient treatment, only a minority of ccRCC patients can acquire long-lasting benefits due to the heterogeneity of the TME (14). The development of ccRCC was also shown to be associated with metabolism, and many mutated genes were found to have roles in metabolic pathways (15). The depletion of a gluconeogenic enzyme—fructose-1,6-bisphosphatase 1 (FBP1)—promoted cell proliferation and glycolysis, associating with worse prognosis of ccRCC patients (16). Moreover, alterations in lipid metabolism process were also involved in progression of ccRCC. The overexpression of fatty acid synthase was associated with poor prognosis of ccRCC patients (17). Different metabolism signatures may cause prognosis heterogeneity in ccRCCs, suggesting the possibility to classify ccRCCs from metabolic perspective. To date, variable immune-related classification methods based on gene signatures or immune components in transcriptome studies showed prediction values in ccRCC immunotherapy (18–20). However, the interplay of immune activity with metabolism is crucial for the regulation of the TME network. For example, tryptophan catabolism was reported to be highly correlated with immune suppression in renal cell carcinoma compared with other metabolic process (21). Therefore, integrating immune activity and metabolism into the classification system provides new insights into discovering reliably predictive biomarkers for ICI therapy.

To date, comprehensive transcriptomic analysis that evaluates the pattern of interaction occurring between metabolism and immune activity in ccRCC remains rare. Here, to dissect the complexity of the interaction between metabolism and immune system in ccRCC, we conducted comprehensive transcriptomic analysis and identified four subtypes with distinct immune and metabolic characteristics. The exploration of the dynamic shift in immunometabolism patterns after immunotherapy provides new clues for the prediction of immunotherapy response. Using multiomics data, we also deciphered the genetic and epigenetic mechanisms that might lead to biological discrepancies among the four subtypes. In addition, we attempted to determine whether the preoperative imaging features could reveal valuable information for the non-invasive prediction of the immune and metabolic status of ccRCC.

## Materials and Methods

### Data Acquisition and Preprocessing

Two transcriptomic datasets of ccRCC and matched normal kidney tissues named TCGA-KIRC (The Cancer Genome Atlas, Kidney Renal Clear Cell Carcinoma) (tumor sample, T=525; normal sample, N=72) and GSE53757 (T=72, N=72) were utilized to identify metabolism-related genes. Moreover, GSE15641 (T=32, N=23) and GSE66272 (T=27, N=27) were utilized as validation sets to evaluate the discriminative capacity of candidate metabolism genes. Tumor samples with patient survival information in TCGA-KIRC (n=513) and RECA-EU cohort from International Cancer Genome Consortium (ICGC) (n=92) were included in our study for the identification and verification of ccRCC subtypes, respectively. Additionally, we also collected transcriptomic data of responders and non-responders to anti-PD-1 therapy, including bladder cancer (IMvigor210, n=298), ccRCC [Braun et al. (22), n=16], and melanoma (GSE91061, n=43) patients. Patients with missing response status information were excluded. GSE64052, containing five sunitinib-sensitive samples and four sunitinib-resistant samples, was included for correlation analysis. All datasets utilized in our study are summarized in **Supplementary Table S1**. For the microarray dataset downloaded from Gene Expression Omnibus (GEO), the raw CEL files were normalized independently using the robust multiarray average method with the “affy” R package. All the transcript data were transferred to HUGO Symbols, and the gene expression data quantified as fragments per kilobase million (FPKM) or read counts were converted to transcripts per million (TPM). When necessary, the TPM value was transformed into log2(TPM+1).

Multiomics data, including miRNA expression, copy number variations (CNVs), somatic mutation, and DNA methylation data, were downloaded from the TCGA database and UCSC Xena website. Somatic mutation data were processed using VarScan software (http://varscan.sourceforge.net/). The “maftools” R package was implemented to analyze and visualize the mutation annotation format (MAF) of somatic variants. Tumor mutation burden (TMB) was calculated as the number of variants divided by the length of exons (38 million) for each sample.

Contrast-enhanced CT scans from treatment-naive ccRCC patients were collected from the Cancer Imaging Archive (TCIA) (n=133) and Zhongshan Hospital (n=45). Only images from the arterial phase were implemented for the extraction of radiomic features. Ethical approval was confirmed by the Zhongshan Hospital Research Ethics Committee.

### Identification of Metabolism-Related ccRCC Subtypes

Genes enriched in the 186 metabolism-related Kyoto Encyclopedia of Genes and Genomes (KEGG) pathways were obtained from the Molecular Signature Database v7.1 (MSigDB). Metabolism-related genes were identified after duplicate deletion for subsequent consensus clustering. Before consensus clustering, there was a three-step strategy for selecting candidate genes. First, 940 genes from TCGA-KIRC and 864 genes from GSE53757 were identified. Second, the abovementioned genes were used as separate input gene sets for weighted gene coexpression network analysis (WGCNA) using the “WGCNA” R package to determine which gene module was most relevant to ccRCC or normal kidney tissue. Meanwhile, the metabolism-related genes differentially expressed (DE-MRGs) between ccRCC and normal kidney samples were screened with a filtering criteria of a false discovery rate (FDR) q-value <0.05. The genes overlapping at the intersection of the “WGCNA-identified gene module from TCGA,” “WGCNA-identified gene module from GEO,” “DE-MRGs from TCGA,” and “DE-MRGs from GEO” were identified. Finally, univariate Cox regression analysis was conducted to filter prognostic candidate genes with p < 0.05 for clustering. We used consensus clustering with the “ConsensusClusterPlus” R package of iterations of 50 and a resampling rate of 0.8. The optimal k-value was determined to obtain robust clusters. We selected two novel multiomics data subtyping methods, namely, Subtyping Multiomics using a Randomized Transformation (SMRT) (23) and Neighborhood-based Multi-Omics clustering (NEMO) (24), to validate the classification results generated by consensus clustering in TCGA-KIRC cohort.

### Landscape of Immune Infiltration in ccRCC

We utilized three methods, namely, single sample gene-set enrichment analysis (ssGSEA), microenvironment cell population-counter (MCP-counter), and CIBERSORT, to estimate the absolute abundance of immune cells, stromal cells, and immune functions based on the gene expression profile. Using the gene sets including 782 genes for predicting the abundance of 29 immune cell types (http://software.broadinstitue.org/gsea/msigdb/index.jsp), a total of 29 immune cell types and corresponding immune infiltration levels were obtained for each sample after conducting the “Gene Set Variation Analysis (GSVA)” R package. We used the “MCPcounter” R package to confirm the robustness of ssGSEA with the quantification of eight immune and two stromal cell types. CIBERSORT was another computational method applied in our study to calculate the components of macrophages M1 and macrophages M2. In addition, to obtain an overview of the components of the tumor microenvironment, we applied the ESTIMATE algorithm to calculate immune and stromal scores in each tumor sample. Based on the results calculated with ssGSEA, consensus clustering was applied for the identification of immune clusters. SMRT and NEMO were also applied to validate the classification results.

### KEGG Pathway Enrichment Analysis and Gene Set Variation Analysis

KEGG pathway enrichment analysis was performed to analyze the biological functions of differentially expressed genes *via* the “clusterProfiler” R package. An adjusted *p*-value <0.05 was set as the cutoff value. Normalized enrichment scores were calculated for metabolism-related KEGG pathways using the “GSVA” R package. Differential analysis was implemented between different clusters using the “limma” R package with an adjusted *p*-value <0.05 considered differentially enriched pathways.

### Radiomics Analysis

We excluded ccRCC samples with missing patient follow-up information, arterial enhanced phase CT scan data, and poor imaging quality. Consequently, 133 ccRCC samples from TCGA database and 45 ccRCC samples from our institution were enrolled in our study. The tumor masses were manually segmented on the arterial phase of CT scans by two experienced radiologists (C. Y. and C-WZ) with ITK-SNAP software (Version 3.8). All segmentations were validated by a senior radiologist (C.W.Z. with over 15 years of working experience). Feature extraction was conducted on the uAI Research Portal Platform, which was implemented by the Python programming language (version 3.7.3, https://www.python.org), and the “PyRadiomics” R package was embedded into this platform. A total of 2,600 radiomic imaging features were defined to reflect tumor characteristics. Radiomics categories mainly included shape and size, first-order statistics, gray-level co-occurrence matrix, wavelet features, etc. The detailed radiomic features for all patients are presented in **Supplementary Table S2**.

For feature selection, we first normalized radiomic features by Z score for further analysis. To construct a prognostic radiomic score (PRS) model for OS, univariate Cox regression was applied for initial feature selection in TCGA-KIRC (discovery cohort). The radiomic features with FDR <0.05 were included as the input for least absolute shrinkage and selection operator (LASSO) regression with the “glmnet” R package, and “Cox” was set as the family in this algorithm. We used data from an external cohort (Zhongshan cohort, n=45) as the validation cohort for PRS. The LASSO regression method was also used for predicting the metabolic and immune status for samples in TCGA, and “binomial” was set as the family in this algorithm. A total of 133 patients were randomly assigned to the training or validation cohort at a ratio of 7:3. Tenfold cross-validation was performed to obtain the resulting features under the condition of the lambda minimum. The coefficients for the final selected features were used for calculating the PRS for each patient. The formula for calculating the PRS is:


$$PRS=\sum_{i=1}^{n}Coefi*Xi$$

where *Coefi* refers to the coefficient of each selected radiomic feature, and *Xi* refers to the radiomic value after normalization.

### Immunofluorescence

The slides were incubated with TIM-3 antibody (Abcam: ab241332) and GlUT1 antibody (Abcam: ab115730) at 4℃ overnight. Then, the samples were incubated with appropriate rabbit/mouse secondary antibodies (Yeason, Shanghai, China) for 2 h. The nuclei were counterstained with 4′,6-diamidino-2-phenylindole (DAPI) (Yeason, Shanghai, China). The positively stained cells were visualized and counted under a Nikon microscope (200× magnification).

### Additional Bioinformatic and Statistical Analyses

The survival analyses were performed in our study using the “survminer” and “survival” R packages. The “surv_cutpoint” function of the “survminer” R package was applied to determine the best cutoff value on the basis of the maximal log-rank statistics. Survival differences were compared by the log-rank test, and survival curves were drawn by the Kaplan–Meier method. The time-dependent receiver operating characteristic (ROC) curves were plotted to compare the predictive value for survival among different variables using the “timeROC” R package. R packages and software mainly utilized for analyses in our study are summarized in **Supplementary Table S3**. The Spearman correlation test was used to evaluate the relationship between two variables. Student’s *t*-test and Wilcoxon rank-sum test were used to compare the data of two groups with normally distributed and nonnormally distributed variables, respectively. ANOVA and Kruskal–Wallis tests were applied to compare the data of more than two groups with normally distributed and non-normally distributed variables, respectively. A two-tailed *p*-value <0.05 was considered statistically significant.

## Results

### Two Distinct Metabolism Clusters Were Identified With DE-MRGS

**Figure 1:**
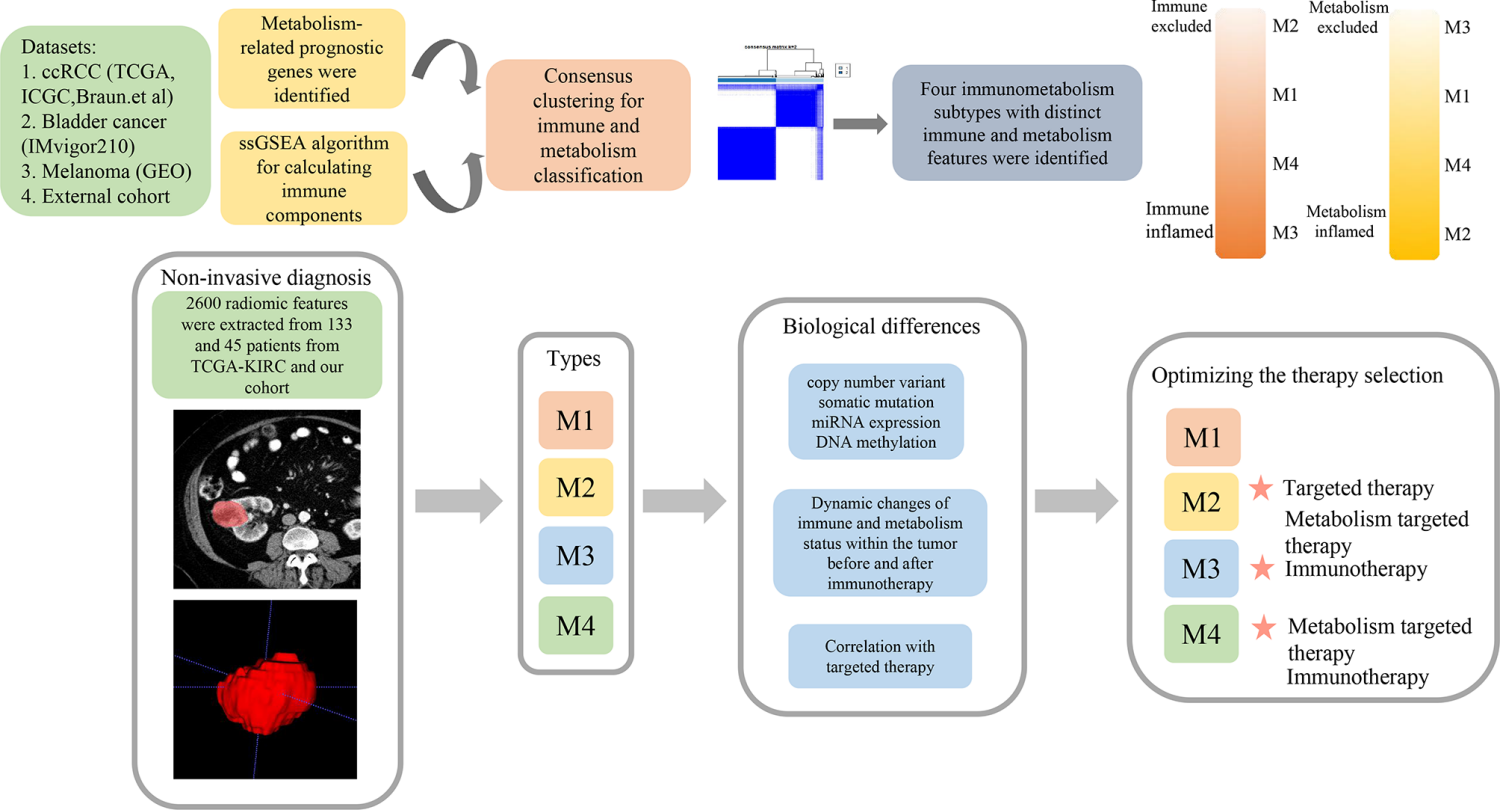
The schematic diagram of the study process.

**Figure 2:**
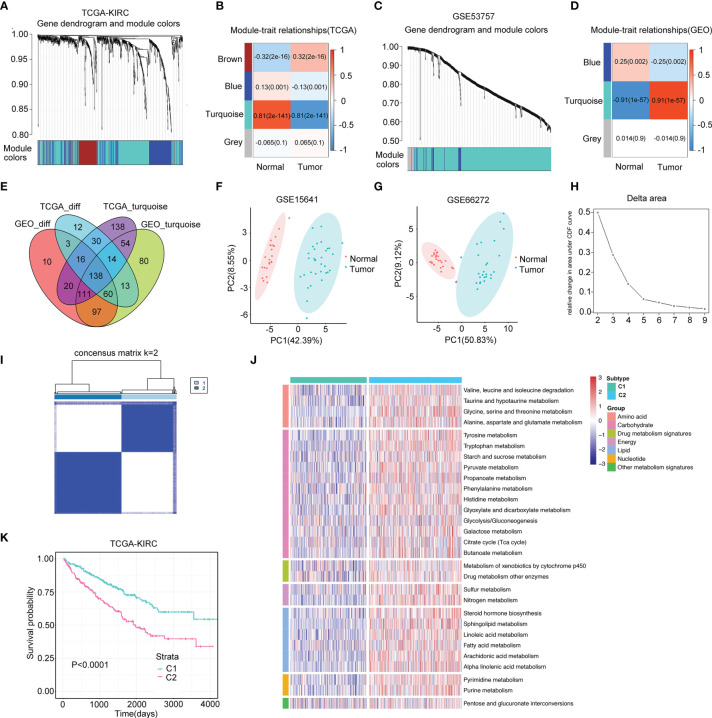
Two distinct metabolism subtypes are identified. **(A)** The weighted gene co-expression network analysis (WGCNA) is conducted with metabolism-related genes in TCGA-KIRC. **(B)** Four gene modules are generated, and the turquoise module presents the highest correlation with sample type. **(C)** WGCNA is conducted with metabolism-related genes in GSE53757. **(D)** Three gene modules are generated, and the turquoise module presents the highest correlation with sample type. **(E)** One hundred thirty-eight overlapping genes in the intersection of the four gene modules are identified for further univariate Cox analysis. **(F, G)** Principle component analyses (PCAs) are performed for another two datasets (GSE15641 and GSE36895), and 56 DE-MRGS can clearly separate tumor samples with normal samples. **(H, I)** The cumulative distribution function curves of consensus matrix indicate that when k=2, the interference between subgroups is minimal. **(J)** Heatmap of the seven categories of metabolism pathways for two subtypes (C1 and C2). **(K)** Survival analysis between two metabolism subtypes. *p*-value is given by log rank test.

A flow chart was plotted to describe the design of our study (**Figure 1**). A total of 4,888 metabolism-related genes were extracted from all metabolism pathways downloaded from MSigDB. Next, we used the WGCNA algorithm to mine for the hub genes in the tumorigenesis process of ccRCC from TCGA-KIRC and GSE53757. For TCGA-KIRC, 525 tumor samples and 75 normal samples were used to construct a scale-free co-expression network. Four gene modules with different colors were generated when the optimal soft threshold was determined to be 3. Among them, the turquoise module with 521 hub genes had the highest correlation coefficient and a low *p*-value (|Pearson Cor|=0.81, *p*<0.01) (**Figures 2A, B** **)**. For the GSE53757 dataset with 72 tumor samples and 72 normal samples, we selected a power of 4 as a soft threshold and obtained three gene modules. The turquoise module with 567 hub genes showed the highest correlation with sample type (|Pearson Cor|=0.91, *p*<0.01) (**Figures 2C, D** **)**. In addition, 286 and 455 metabolism-related genes differentially expressed between tumor and normal samples were identified separately in TCGA-KIRC and GSE53757, respectively. As a result, 138 genes overlapping at the intersection of these four gene modules were obtained (**Figure 2E**). To further filter for the metabolism-related genes with prognostic value, 56 genes were considered ccRCC-specific DE-MRGS with univariate Cox regression analysis (**Supplementary Table S4**). To evaluate the discriminative ability of these 56 prognostic metabolism-related genes for ccRCC, we conducted principle component analyses (PCAs) in GSE15641 and GSE66272, and we distinctly separated different sample categories (**Figures 2F, G** **)**.

A total of 525 samples from TCGA-KIRC were classified according to the expression profiles of these 56 DE-MRGS using consensus clustering. The cumulative distribution function curves of the consensus matrix indicated that when k=2, the interference between subgroups was minimal, and the distribution was significant (**Figures 2H, I** **)**. To validate the stability of such classification, we performed another independent analysis using the RECA-EU cohort. Consistently, two distinct subtypes were still the optimal choice for metabolism-correlated ccRCC classification (**Supplementary Figures S1A, B** **)**. To decipher the landscape of the metabolic processes of the two clusters, we calculated GSVA scores of different metabolic pathways with seven categories, including amino acid, carbohydrate, drug metabolism, energy, lipid, nucleotide, and other metabolism signatures. We found that C2 had obviously higher metabolic activities than that of C1 for samples in both the TCGA-KIRC and RECA-EU cohorts, which are presented in the heatmaps (**Figure 2J** and **Supplementary Figure S1C**). We defined C1 as the metabolism-excluded subtype and C2 as the metabolism-high subtype. Notably, a significantly worse prognosis was observed in C2 than in C1 (**Figure 2K**; **Supplementary Figure S1D**), implying the clinical value of metabolism-related classification.

### Immune Regulation Interacts With Metabolism

**Figure 3:**
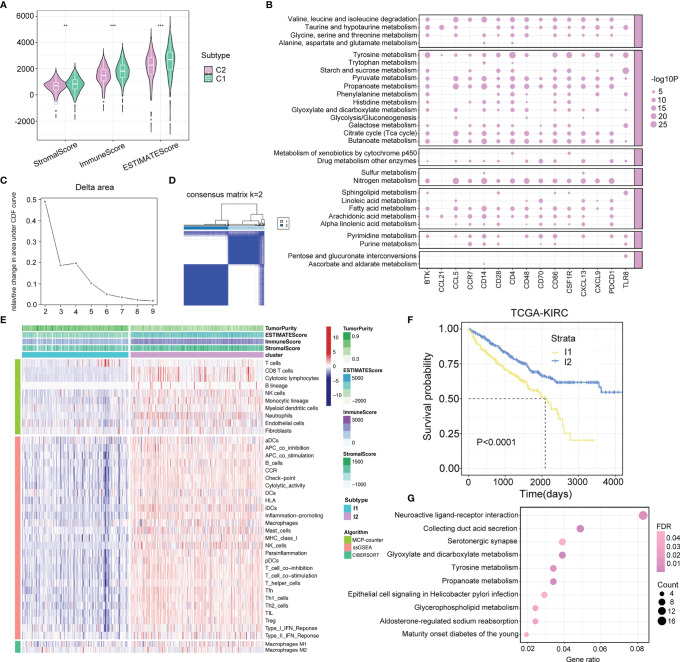
Crosstalk between metabolism and immune regulations. **(A)** Comparison of immune score, stromal score, and estimate score between two metabolism subtypes. p-value is given by Wilcoxon rank-sum test. **p < 0.01, ***p < 0.001. **(B)** The interplay between upregulated immune-related molecules and metabolism pathways. The point represents that the upregulated immune-related molecules are significantly enriched in the relevant metabolism pathways. The size of the point represents the value of −log10 (FDR). **(C, D)** The cumulative distribution function curves of consensus matrix indicate that when k=2, the interference between subgroups is minimal. **(E)** Heatmap describes the abundance of immune components infiltration calculated by ssGSEA, MCP-counter, and CIBERSORT algorithms in two immune subtypes (I1 and I2). **(F)** Survival analysis in I1 and I2 subtypes. *p*-value is given by log rank test. **(G)** Pathway analysis of differentially expressed genes between two immune subtypes.

The dynamic change in the TME is the result of continuous interactions among cancerous cells, non-cancerous cells, and metabolites, which play a decisive role in tumor progression (25, 26). Here, we used the ESTIMATE algorithm to calculate the immune, stromal, and ESTIMATE scores of metabolism clusters, and significant differences were observed between C1 and C2. Intriguingly, C2 showed lower scores than C1 (**Figure 3A**). Moreover, 15 upregulated immune-related molecules in ccRCC samples were enriched in multiple metabolism processes (**Figure 3B**). The above evidence implied that it was of high importance to explore the potential crosstalk pattern between immune and metabolic activity.

To characterize the immune components in the TME of ccRCC, ssGSEA, MCP-counter, and CIBERSORT algorithms were applied to calculate the abundance of immune-related cell types. Based on the ssGSEA results, we recognized two distinct immune subtypes of the TCGA-KIRC cohort using consensus clustering and validated its rationality in the RECA-EU cohort (**Figures 3C, D**; **Supplementary Figures S2A, B** **)**. According to the discrepancy in the immune landscape exhibited between the two subtypes, we defined I1 as the immune-deserted subtype and I2 as the immune-inflamed subtype (**Figure 3E**; **Supplementary Figure S2C**), and I2 patients showed a favorable prognosis (**Figure 3F**; **Supplementary Figure S2D**). Afterward, we performed KEGG pathway analysis for the genes differentially expressed between the two immune clusters. The results showed that a total of 2,782 identified genes were enriched in varying metabolic biological pathways, including glyoxylate and dicarboxylate metabolism, tyrosine metabolism, propanoate metabolism, and glycerophospholipid metabolism (**Figure 3G**).

### Identification of Immunometabolism Subtypes

**Figure 4:**
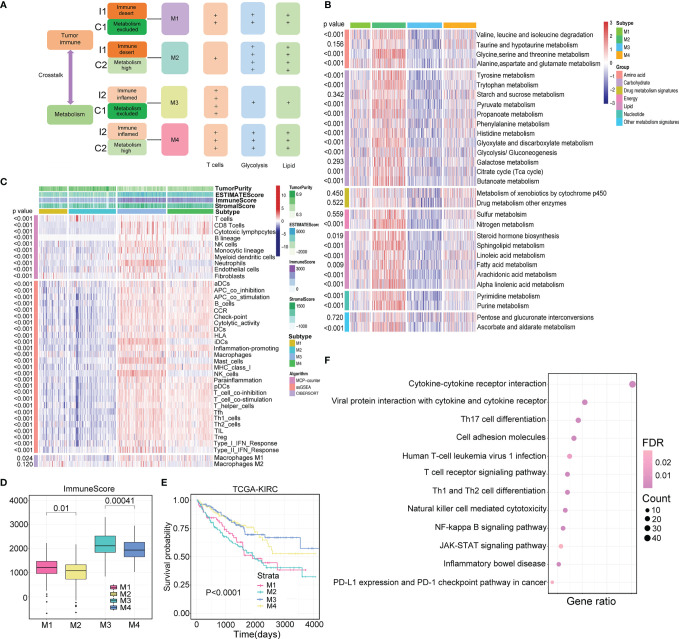
Identification of four immunometabolism subtypes. **(A)** The diagram of immunometabolism classification strategy. **(B**, **C)** Heatmaps of metabolism- and immune-related signatures in four immunometabolism subtypes (M1, M2, M3, and M4). *p*-value is calculated by ANOVA test. **(D)** Comparison of immune score and stromal score in four subtypes. *p*-value is calculated by ANOVA test. **(E)** Comparison of survival outcomes among four subtypes. *p*-value is given by log rank test. **(F)** Specific differentially expressed genes in M2 subtype compared with other subtypes involve in multiple immune relevant pathways.

Considering the synergistic effect of immune activity and metabolism, we redefined immunometabolism clusters according to the above immune and metabolism clustering results. Since ccRCC could be classified into two clusters based on immune or metabolism characteristics, four subtypes were generated: the M1 subtype with immune- and metabolism-deserted characteristics, the M2 subtype with immune-deserted and metabolism-high characteristics, the M3 subtype with immune-inflamed and metabolism-deserted characteristics, and the M4 subtype with immune-inflamed and metabolism-high characteristics. The classification scheme is presented in **Figure 4A**. SMRT and NEMO clustering methods were performed to validate the results of immune and metabolism classifications generated by consensus clustering in TCGA-KIRC cohort, respectively. Samples could be mainly classified into two immune or metabolism subtypes (Cluster 1-1 and Cluster 1-2) using SMRT (**Supplementary Figures S3A, B** **)**. PCA plots also showed that samples classified into two immune or metabolism subtypes by NEMO could be separated clearly (**Supplementary Figures S3C, D** **)**. The subtypes identified by the three clustering methods had significant overlapping rates (all *p* < 0.05, **Supplementary Figures S3E–G**), suggesting the good consistency between the three clustering methods.

To investigate whether the four subtypes could reflect the interaction of immune and metabolic processes, we drew heatmaps reflecting the metabolic and immune landscapes in the TCGA-KIRC cohort (**Figures 4B, C** **)** and compared the GSVA score and immune infiltration level of each type (**Supplementary Table S5**). We found that the M2 subtype exhibited the highest metabolic activity, which was significantly higher than that of the M4 subtype. Moreover, the M3 subtype showed the lowest metabolic activity, which was significantly lower than that of the M1 subtype. On the other hand, the M3 subtype had the highest level of immune infiltration, which was significantly higher than that of the M4 subtype, and the M2 subtype had the lowest level of immune infiltration, which was significantly lower than that of the M1 subtype. The above results were also demonstrated in the RECA-EU cohort (**Supplementary Figures S4A, B**; **Supplementary Table S5**). Significantly different immune scores were observed among the four subtypes, with higher immune scores for M1 than M2 (*p*=0.01) and higher immune scores for M3 than M4 (*p*<0.01) (**Figure 4D**). Based on the above evidence, we envisioned that high metabolic activity in the TME can suppress immune activity to some extent, thereby promoting tumor development. The prognostic value of immunometabolism subtype classification was assessed in TCGA-KIRC, and patients of the four subtypes displayed dramatic prognostic differences (log-rank test *p*<0.01, **Figure 4E**). The same result was observed in the RECA-EU cohort (log-rank test *p*<0.01, **Supplementary Figure S4C**). Notably, in the RECA-EU cohort, patients classified as M2 had the worst survival outcome, which was significantly inferior to that of M1 (log-rank test *p*=0.036). Furthermore, similar immune and metabolism patterns of the four clusters in TCGA-KIRC cohort generated by SMRT and NEMO clustering methods were observed compared with consensus clustering (**Supplementary Figures S5A–D**). Increasing evidence has demonstrated that if tumor cells consume increasing amounts of glucose, then T cells would be more metabolically restricted. Therefore, we estimated that the M2 subtype might display the most malignant characteristics. We further explored which biological pathways that the genes underlying the M2 subtype participated in. Interestingly, M2-specific genes were mainly enriched in immunological pathways (**Figure 4F**). Immunometabolism Subtype Classification Correlates With Immunotherapy Efficacy

**Figure 5:**
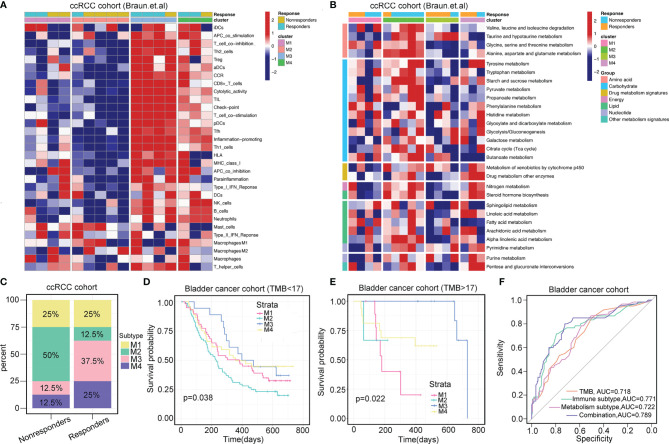
Immunometabolism subtypes correlate with immunotherapy. **(A, B)** Heatmaps show the immune landscape and metabolism signatures of four immunometabolism subtypes in ccRCC prior to anti-PD-1 therapy (Baun et al.). **(C)** Percentages of four immunometabolism subtypes in responders and non-responders prior to anti-PD-1 therapy in ccRCC. **(D, E)** Stratification survival analysis of TMB <17 and TMB >17 across four subtypes in bladder cancer (IMvigor210 cohort). *p*-value is given by log-rank test. **(F)** Receiver operating characteristics of predicting the response of anti-PD-1 therapy in bladder cancer based on the TMB and immunometabolism subtypes.

In the ccRCC cohort (Braun et al.) and bladder cancer anti-PD-1 therapy cohort (IMvigor210), we classified patients into four subtypes that showed consistent patterns of immune and metabolic activities (**Figures 5A, B**; **Supplementary Figures S6A, B** **)**. The response rate of patients classified by the four subtypes was determined. In the ccRCC cohort, the overall response rate was significantly higher in patients with the M3 subtype (37.5%) than in those with the M4 subtype (25%) (**Figure 5C**). Similarly, in the bladder cancer cohort, patients classified with the M3 subtype (47.1%) had significantly higher response rates than the other subtypes (**Supplementary Figure S6C**).

TMB is the most prevalent biomarker for predicting immunotherapy response. We attempted to investigate whether this immunometabolism classification system can be utilized as a robust tool to predict the responses to immunotherapy compared with that achieved using TMB. Next, we determined the cutoff point of TMB as 17 using the “surv_cutpoint” function of the “survminer” R package. For samples in the TMB <17 group or TMB >17 group, similar results were observed, with prolonged OS associated with M3 subtype classification and dismal OS associated with M2 subtype classification (log-rank test, *p*=0.038 for TMB <17, and log-rank test, *p*=0.022 for TMB <17, **Figures 5D, E** **)**. This TMB stratification survival analysis indicated that immunometabolism classification had a stable and independent capacity for predicting OS. Importantly, combining TMB status with the classification of immunometabolism improved the predictive accuracy of TMB and metabolism classification but was non-inferior to that achieved using immune classification (TMB vs. TMB + immunometabolism classification, *p*=0.030; TMB vs. metabolism classification, p=0.025; TMB vs. immune classification, p=0.783, **Figure 5F**). Overall, the immunometabolism classification system can complement TMB as a predictor for immunotherapy response.

### The Dynamic Pattern of Changes to the Immune Landscape During Immunotherapy

**Figure 6:**
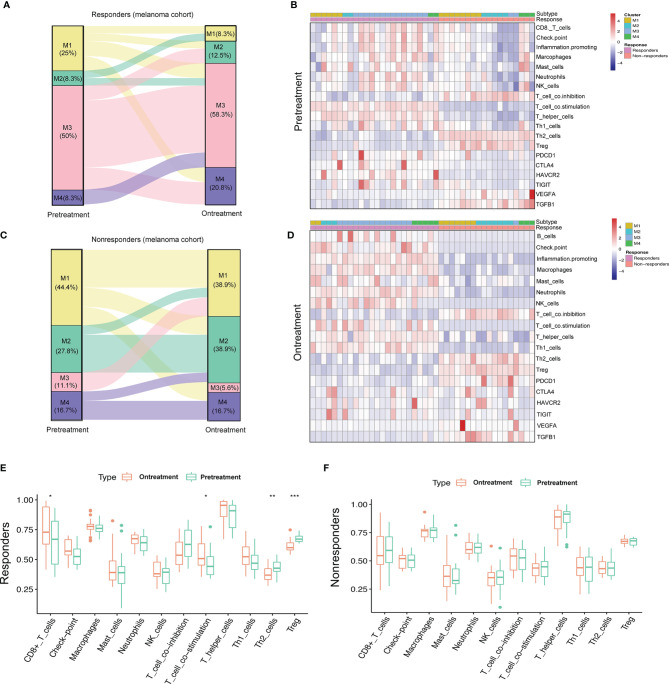
Dynamic change pattern of immunometabolism subtypes during the immunotherapy. **(A, B)** Sankey diagram shows the flow change of immunometabolism subtypes of the responders (n=24) and non-responders (n=18) to anti-PD-1 therapy pretreatment and on-treatment. **(C, D)** Heatmaps show the immune components and immune relevant molecules of patients pretreatment and on-treatment. **(E, F)** Boxplots compare the differences of immune components pretreatment and on-treatment. p-value is given by Wilcoxon rank-sum test. *p < 0.05, **p < 0.01, ***p < 0.001.

It is crucial to understand the pattern of TME modulation that occurs during immunotherapy since such dynamic changes can reflect the mechanism of resistance to immunotherapy. We analyzed the pretreatment and on-treatment transcriptomic data of a melanoma patient cohort (GSE91061) receiving anti-PD-1 therapy with 24 responders and 18 non-responders. All the samples were classified into four subtypes. The M3 subtype accounted for the largest proportion of patients before treatment (50%) and during treatment (58.3%) in the responder group, while the M2 subtype accounted for the largest proportion of non-responders before treatment (27.8%) and during treatment (38.9%). We observed that in the responder group, samples that were classified into the M1 and M2 subtypes before treatment evolved into active immune groups (two of M1 evolved to M3, two of M1 evolved to M4, and one of M2 evolved to M3, **Figure 6A**), while this evolutionary trend was the opposite in the non-responder group. M1 and M2 classifications mainly retained their observed pretreatment prevalence during the treatment; however, the M3 and M4 subtypes showed a tendency to evolve to exhausted immune groups (two of M3 evolved to M1, one of M3 evolved to M3, **Figure 6B**). The immune profiles of patients in the non-responder group were characterized by higher infiltration levels of immune-suppressive cells, such as Th2 cells and Treg cells, lower expression of immune checkpoint molecules and higher expression of two immunosuppressors (VEGFA and TGFB1) than those of patients in the responder group. The immune profiles of patients before treatment and during treatment are displayed in heatmaps (**Figures 6C, D** **)**. Notably, during treatment, patients from the responder group expressed higher levels of PDCD1 (PD-1), CTLA4, HAVCR2, and TIGIT, implying the efficacy of immunotherapy. Meanwhile, Th2 cells and Treg cells also had significantly lower infiltration levels, while CD8+ T cells infiltrated more (**Figure 6E**). However, no significant difference was observed in the immune profiles for non-responders (**Figure 6F**). In general, our results implied that the reason for immunotherapy resistance was rather complex because the TME condition was dynamic and plastic. Therefore, inducing a shift of the TME with other therapeutic approaches for more favorable responses to immunotherapy is of high value.

### Correlation of the Immunometabolism Subtypes With miRNA Expression, DNA Methylation, CNV, and Mutations

**Figure 7:**
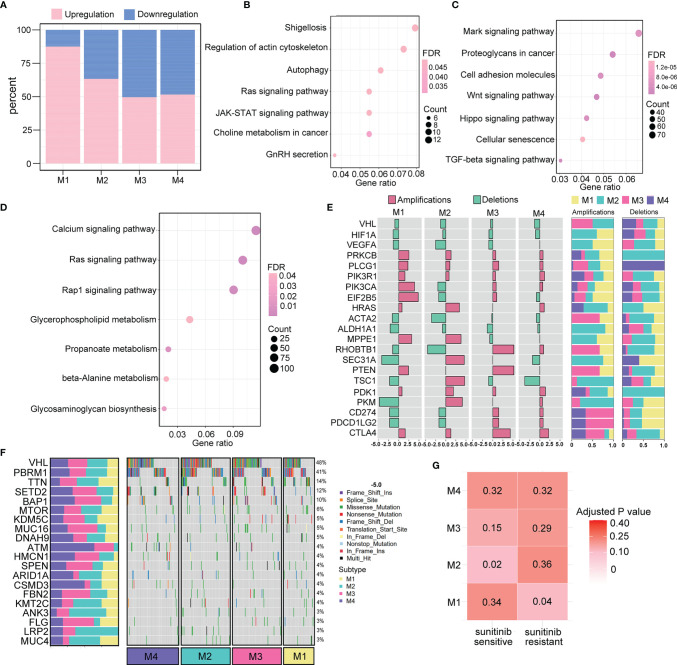
Genomic and epigenomic features of the immunometabolism subtypes. **(A)** Percentage of significantly down- and upregulated miRNAs in TCGA across four subtypes. **(B**, **C)** Pathway analysis of target genes regulated by up- and downregulated miRNAs of M3 subtype. **(D)** Pathway analysis of M1-specific hypermethylated genes. **(E)** Subtype-specific CNV (deletions or amplifications) enrichment is presented as odd ratios with FDR <0.05. The proportion of deletion events and amplification events in the four subtypes are denoted on the right. **(F)** Waterfall of mutation status of the top 20 most mutated genes among the four subtypes. The proportion of mutation rates of the corresponding genes in the four subtypes is denoted on the left. **(G)** Correlation analysis of immunometabolism subtypes and patients who are sensitive or resistant to sunitinib targeted drug therapy.

Genomic and epigenetic alterations were demonstrated to contribute to the underlying biological differences among the four subtypes (27, 28). Therefore, we conducted differential analyses of miRNA expression and DNA methylation by comparing tumor samples of each subtype. With the criteria of log2|FC|>2 and FDR <0.05, a total of 160 significantly differentially expressed miRNAs (20 downregulated and 140 upregulated) were identified for the M1 subtype, 139 for the M2 subtype (51 downregulated and 88 upregulated), 121 for the M3 subtype (61 downregulated and 60 upregulated), and 95 for the M4 subtype (46 downregulated and 49 upregulated). The proportions of upregulated and downregulated miRNAs are presented in the boxplot (**Figure 7A**).

Moreover, a total of 281 differentially expressed methylated genes were identified for the M1 subtype, 2,014 for the M2 subtype, 5,350 for the M3 subtype, and 913 for the M4 subtype. These results indicated that the M1 and M3 subtypes were more likely to be epigenetically modified. KEGG pathway analysis was performed for the M1 subtype, which had the highest number of significantly differentially expressed miRNAs. We found that 20 significantly downregulated miRNAs were enriched in functions related to the cell cytoskeleton and metabolism (**Figure 7B**), and 140 significantly upregulated miRNAs were enriched in multiple cancer-related pathways (**Figure 7C**). Moreover, M3 showed the highest level of methylation, and the differentially methylated genes were enriched in cancer-related pathways and metabolism pathways (**Figure 7D**).

The association of the pattern of CNV in tumorigenic and immunoregulatory genes with four subtypes was explored. We chose one type of copy number event (deletions or amplifications) depending on which type of copy number event occurred more frequently to calculate the odds ratio that represented the enrichment for each subtype (**Figure 7E**). We found that the M3 and M4 subtypes were enriched with CD274 (PD-L1), PD-L2, and CTLA4 gene amplifications, suggesting that their active responses to immunotherapy were partly due to the increased expression of immune checkpoint molecules. Additionally, several metabolism-related genes, such as PDK1 and PKM, were significantly amplified in the M2 subtype. These findings were consistent with the immunometabolism characteristics of each subtype, providing opportunities to explore new drugs targeting crucial proteins involved in metabolic pathways. Furthermore, the top 20 gene mutation frequencies among the four subtypes were identified (**Figure 7F**). The loss of VHL, which is the most commonly mutated gene in ccRCC, can result in the upregulation of hypoxia-responsive genes, which can induce tumorigenesis. We found that VHL was less frequently mutated in the M1 subtype, which indicates the probability of resistance to targeted therapy. Indeed, in further correlation analysis of our four subtypes with the drug sensitivity dataset, M1 subtype classification showed a significant correlation with TKI resistance (*p*=0.04), whereas M2 subtype classification was correlated with TKI sensitivity (*p*=0.02) (**Figure 7G**).

### Radiomic Profile Predicts Survival and Reflects Status of the Immune System and Metabolism

**Figure 8:**
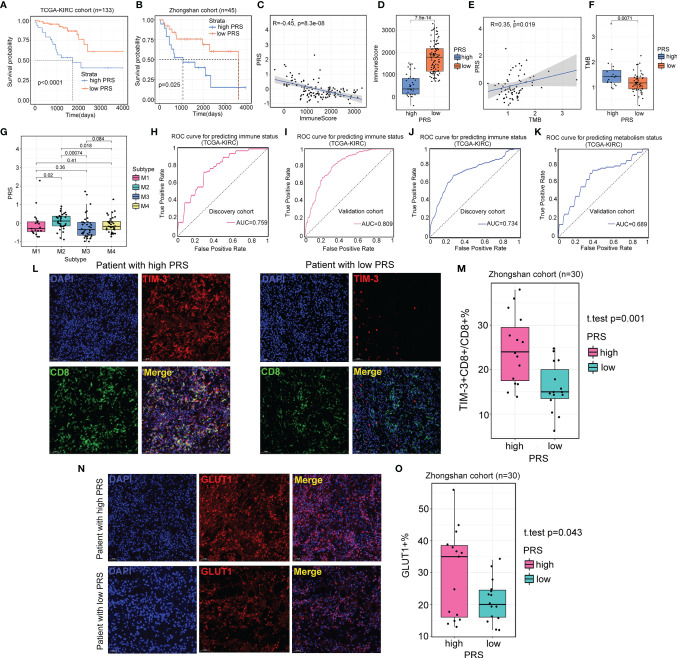
Radiomics profile reveals the status of immune and metabolism. **(A, B)** Survival analysis of patients from different prognostic radiomic score (PRS) groups in the TCGA-KIRC cohort (n=133) and Zhongshan cohort (n=45). **(C, D)** Scatter plot and boxplot show the correlation between PRS and immune score. For scatter plot, *p*-value is given by the Spearman correlation analysis. For boxplot, *p*-value is given by Wilcoxon rank-sum test. **(E, F)** Scatter plot and boxplot showing the correlation between PRS and TMB. For scatter plot, *p*-value is given by the Spearman correlation analysis. For boxplot, *p*-value is given by Wilcoxon rank-sum test. **(G)** The association between PRS and four immunometabolism subtypes. *p*-value is given by Kruskal–Wallis test. **(H, I)** Results of area under the curve (AUC) for the 10 radiomic features predicting immune status in the discovery cohort (TCGA-KIRC cohort, n=91) and validation cohort (TCGA-KIRC cohort, n=42). **(J, K)** Results of AUC for the 11 radiomic features predicting immune status in the discovery cohort (TCGA-KIRC cohort, n=91) and validation cohort (TCGA-KIRC cohort, n=42). **(L)** Representative images of the immunofluorescence staining with TIM-3 (red), CD8 (green), DAPI (blue), and Merge (double positive) on ccRCC tissues and their paired normal tissues from high and low PRS groups. Scans are imaged at 200 magnification. **(M)** The percentage of TIM-3 expressed on CD8^+^ T cells is higher in high PRS group (n=15) than low PRS group (n=15). *p*-value is given by Student’s t-test. **(N)** Representative images of the immunofluorescence staining with GLUT1 (red), DAPI (blue), and Merge on ccRCC tissues and their paired normal tissues from high and low PRS groups. Scans are imaged at 200 magnification. **(O)** The percentage of the expression of GLUT1 is more in high PRS group (n=15) than low PRS group (n=15). *p*-value is given by Student’s t-test.

We utilized two independent datasets to evaluate the prognostic value of the radiomic profile for ccRCC patients: the TCGA-KIRC cohort as the discovery cohort (n=133) and the Zhongshan cohort as the validation cohort (n=45). We first conducted univariate Cox regression analysis for 2,600 radiomic features in the TCGA-KIRC cohort, and 16 radiomic features were determined to be significantly associated with OS (FDR<0.05). Then, the 16 radiomic features were further filtered and reduced to 7 radiomic features using LASSO regression. The survival information and the seven radiomic profiles for the two cohorts are presented in **Supplementary Table S6**. A PRS was calculated for each sample. Using the “surv_cutpoint” function of the “survminer” R package, we determined 0.121 to be the cutoff PRS to stratify patients into two groups (high PRS, n=35; low PRS, n=98). The OS difference was observed in the different risk groups in the TCGA-KIRC cohort (log-rank test, *p*<0.01, **Figure 8A**) and confirmed in the Zhongshan cohort, with 26 patients stratifying into the low PRS group and 19 patients stratifying into the high PRS group (log-rank test, *p*=0.025, **Figure 8B**). When investigating the biological characteristics of patients classified into different PRS groups, we noted that a higher PRS was inversely related to a lower immune score (**Figures 8C, D** **)**. In contrast, the PRS was positively associated with the TMB score (**Figures 8E, F** **)**. In addition, we observed that patients classified into the M2 subtype had a significantly higher PRS than those of the M1, M3, and M4 subtypes (**Figure 8G**), which was consistent with the finding that the worst OS was observed in the patients with M2 subtype. Considering that the PRS was associated with immunometabolism subtype classification, we further explored the predictive value of radiomic profiles for immune and metabolism status. For immune status prediction, 10 radiomic features were selected by LASSO regression (**Supplementary Table S7**). The prediction model achieved areas under the curve (AUCs) of 0.759 and 0.805 for the discovery and validation cohorts, respectively (**Figures 8H, I** **)**. Moreover, the prediction model comprised 11 radiomic features (**Supplementary Table S8**), and classification by metabolism status obtained AUCs of 0.689 and 0.743 for the discovery and validation cohorts, respectively (**Figures 8J, K** **)**.

Additionally, to confirm the relationship of PRS with immune and metabolic status, we examined the expression of TIM-3^+^CD8^+^ T cells and GLUT1^+^ cells from tumors and paired normal tissues from patients in the high PRS group (n=15) and low PRS group (n=15). The number of TIM-3^+^CD8^+^ T cells was higher in the high PRS group (*p*=0.001, **Figures 8L, M** **)**. Patients from the high PRS group also expressed significantly higher levels of GLUT1 than those of their counterparts (*p*=0.043, **Figures 8N, O** **)**. These results confirmed that patients with a high PRS tended to have suppressive immune activity and active metabolic activity.

## Discussion

In this study, we aimed to explore the synergistic effect of the immune response and metabolism in ccRCC progression to determine whether this effect contributes to the classification of ccRCC. We first separately identified two immune- and metabolism-related classifications of ccRCC utilizing the consensus clustering method. Then, based on the results of the initial classifications, we redefined four subtypes of ccRCC, namely, M1, M2, M3, and M4, which could reflect the interplay between immunity and metabolism. Generally, we found that a high metabolic state in the TME could decrease immune cell infiltration. Importantly, this combined classification was significantly correlated with patient OS. Patients with the M2 subtype harbored an inflamed metabolic state, possessed reduced immune activity, and exhibited the worst OS, while patients with the M3 subtype had the exact opposite features of those of the M2 subtype and exhibited the best OS.

We explored the genetic aberrations and epigenetic mechanisms underlying the heterogeneity of these four subtypes. Notably, the M2 subtype classification, which exhibited relatively higher frequencies of VHL mutations, was also associated with sensitivity to sunitinib therapy, but this fraction of patients with immune-excluded characteristics tended to be less responsive to immunotherapy in our study. Recently, investigations have demonstrated that the combination of ICI therapy and VEGF TKI therapy can increase the response rate and overall survival of ccRCC patients (29, 30). Interestingly, there was a finding suggesting that targeting the VEGF pathway could suppress the influx of suppressive immune cells into the TME (31–33). This finding indicated that VEGF TKI therapy served as an advantageous combination with ICI therapy. Our data suggested that copy number changes might also have an effect on the immunotherapy response. CD274 (PD-L1) and PDCD1LG2 (PD-L2) gene amplifications were enriched in the M3 and M4 subtypes, suggesting a higher expression of immune checkpoint molecules and better efficacy of ICI therapy for the treatment of patients with M3 and M4 subtypes than for patients with the other two subtypes. Concordant with the above results, a similar trend was also demonstrated in melanoma and bladder cancer ICI therapy datasets. Further clinical trials are required to test the efficacy of the combination of ICI therapy and targeted therapy for patients with the M2 subtype.

ICI therapy can reprogram the tumor immune microenvironment in ccRCC patients by upregulating the expression level of the coinhibitory receptors and effector molecules of cytotoxic T cells (34). In addition, the findings of several studies indicated that ICI therapy can have the potential to influence the cross-communication between tumors and T cells. The inhibition of the PD-1-PD-L1 axis in tumor cells can dampen aerobic glycolysis *via* suppression of the PI3K–AKT–mTOR pathway (35–37). As a result, the function of TILs would be restored with the increase in available glucose. Similarly, in our study, we observed that there were no evident changes in the immune landscape in the ICI-resistant group before and after therapy. However, for the responsive group, the TME of ccRCC patients after therapy was characterized by significantly more effector T cells and fewer suppressive T cells. Samples from the responder group tended to transfer to subtypes with immune-inflamed characteristics and less active metabolic activity. These findings indicated that immunity and metabolism, the two key elements influencing the TME, were independently linked with each other. Moreover, since accumulating evidence has demonstrated that targeting metabolic pathways in tumors with inflamed metabolism could convert the resistance to immunotherapy (38–40), we speculated that patients with the M2 subtype might gain more benefit from ICI therapy accompanied by metabolism-targeted therapy. Nonetheless, our analysis was based on bulk sequence data, which cannot reflect specific cell-type patterns. The proportion of activated CD8^+^ T cells was strongly associated with the prognosis of ccRCC patients in multiple studies (41, 42). Bi et al. (34) found that macrophages of ccRCC patients shifted toward proinflammatory states but also exhibited upregulated expression of immunosuppressive markers during immunotherapy. In the future, single-cell sequencing combined with multiomics approaches will provide deeper insights into the reprogramming of immune- and metabolism-related processes during immunotherapy.

Currently, the status of the tumor microenvironment can be evaluated only once on histological specimens after surgery, and the assessment accuracy might be limited by the heterogeneity of biopsy. Previous studies have discovered that radiomic features, reflecting subtle homogeneity or heterogeneity utilizing the gray-level run length matrix, are associated with the expression of TILs (43, 44). Radiomic analysis also achieved satisfactory performance for the prediction of clinical outcomes of immunotherapy patients across multiple cancers (45–47). Here, to non-invasively evaluate the immune and metabolic status of ccRCC patients, we built prediction models that demonstrated satisfactory performances. Furthermore, we also developed PRS and validated its prognostic value in our center. Intriguingly, patients with the M3 subtype had the highest PRS and the worst prognosis, which indicated that the PRS could reflect the combined effect of immunity and metabolism to some extent. Our study illustrated the link between imaging features and TME status, which can provide clinicians with meaningful biological information for optimizing treatment strategies. However, further evaluation with a large clinical sample size is warranted to validate our results.

In general, we classified ccRCC patients into four subtypes based on the synergistic effect of immune activity and metabolism. A high metabolic status, especially regarding fatty acid metabolism and glycolysis, suppresses immune cell infiltration, and these processes have a strong association with TILs. We unveiled the molecular differences underlying patients with the four subtypes from both genetic and epigenetic perspectives. We also illustrated the dynamic changes in the immune landscape before and after immunotherapy. In addition, radiomic analysis was demonstrated to have predictive value for immune and metabolic status. These results could provide guidance for ccRCC classification and improve precision diagnosis and treatment of ccRCC patients.

## Data Availability Statement

Publicly available datasets were analyzed in this study. This data can be found here: https://portal.gdc.cancer.gov/, https://www.ncbi.nlm.nih.gov/geo/query/acc.cgi?acc=GSE53757, https://www.ncbi.nlm.nih.gov/geo/query/acc.cgi?acc=GSE15641, https://www.ncbi.nlm.nih.gov/geo/query/acc.cgi?acc=GSE66272, https://www.ncbi.nlm.nih.gov/geo/query/acc.cgi?acc=GSE91061, https://www.ncbi.nlm.nih.gov/geo/query/acc.cgi?acc=GSE64052, https://dcc.icgc.org/.

## Ethics Statement

The studies involving human participants were reviewed and approved by Clinical Research Ethics Committee of Zhongshan Hospital of Fudan University. The patients/participants provided their written informed consent to participate in this study.

## Author Contributions

YW and XZ designed the study. YW, XZ and GZ did the bioinformatic analysis. YW and NL collected the clinical data. YW and CY collected tumor tissues and did the experiment. YW, CZ and NL prepared figures. YW, XZ, GZ, NL, CZ and MZ reviewed the results, interpreted data and wrote the manuscript. All authors have made an intellectual contribution to the manuscript and approved the submission.

## Funding

This work was supported by grants from the National Natural Science Foundation of China (grant number 82171897), Shanghai Science and Technology Committee (grant number 19411965500), Shanghai Municipal Key Clinical Specialty (grant number shslczdzk03202), Clinical Research Plan of SHDC (grant number SHDC2020CR1029B), Clinical Research Project of Zhongshan Hospital, Fudan University (grant number 2020ZSLC61).

## Conflict of Interest

The authors declare that the research was conducted in the absence of any commercial or financial relationships that could be construed as a potential conflict of interest.

## Publisher’s Note

All claims expressed in this article are solely those of the authors and do not necessarily represent those of their affiliated organizations, or those of the publisher, the editors and the reviewers. Any product that may be evaluated in this article, or claim that may be made by its manufacturer, is not guaranteed or endorsed by the publisher.

## Supplementary Material

Supplementary Figure 1 Two distinct metabolism subtypes are validated in RECA-EU cohort. **(a, b)** The CDF curves of consensus matrix indicates that when k=2, the interference between subgroups is minimal. **(c)** Heatmap of the seven categories of metabolism pathways for two subtypes (C1 and C2).**(d)** Survival analysis between two metabolism subtypes. P value is given by log rank test.

Supplementary Figure 2 Two distinct immune subtypes are validated in RECA-EU cohort. **(a, b)** The CDF curves of consensus matrix indicates that when k=2, the interference between subgroups is minimal. **(c)** Heatmap of the aboundance of immune components infiltration for two subtypes (I1 and I2). **(d)** Survival analysis between two immune subtypes. P value is given by log rank test.

Supplementary Figure 3 Immune and metabolism classification results in TCGA-KIRC cohort generated by SMRT and NEMO clustering method show good consistency with consensus clustering. **(a, b)** PCA plots show that samples can be mainly classified into two immune and metabolism subtypes by SMRT clustering method. **(c, d)** PCA plots show that two immune and metabolism subtypes can be separated clearly using NEMO clustering method. **(e-g)** Sankey diagram shows that the three clustering methods, consensus clustering, SMRT and NEMO have significant overlapping samples in immune subtypes **(e)**, metabolism subtypes **(f)** and immunometabolism subtypes **(g)**.

Supplementary Figure 4 Validation of four immunometabolism subtypes in RECA-EU cohort. **(a, b)** Heatmaps of metabolism and immune related signatures in four immunometabolism subtypes (M1, M2, M3 and M4). P value is calculated by ANOVA test. **(c)** Comparison of survival outcomes among four subtypes. P value is given by log rank test.

Supplementary Figure 5 The immune and metabolism landscapes of the four immunometabolism clusters in TCGA-KIRC cohort generated by SMRT and NEMO clustering methods.** (a, b)** Heatmaps of metabolism and immune related signatures in four immunometabolism subtypes (M1, M2, M3 and M4) generated by SMRT clustering method. P value is calculated by ANOVA test. **(c, d)** Heatmaps of metabolism and immune related signatures in four immunometabolism subtypes (M1, M2, M3 and M4) generated by NEMO clustering method. P value is calculated by ANOVA test.

Supplementary Figure 6 Immunometabolism subtypes of bladder cancer correlate with immunotherapy. **(a, b)** Heatmap shows the immune landscape and metabolism signatures of four immunometabolism subtypes in bladder cancer prior to anti-PD-1 therapy (IMvigor210 cohort). **(c)** Percentages of four immunometabolism subtypes in responders and nonresponders prior to anti-PD-1 therapy in bladder cancer.
