# Supplementary figures and images for: Crosstalk Between Metabolism and Immune Activity Reveals Four Subtypes With Therapeutic Implications in Clear Cell Renal Cell Carcinoma
Source: Front Immunol. 2022 Apr 11;13:861328. doi: 10.3389/fimmu.2022.861328 (PMC9035905; doi:10.3389/fimmu.2022.861328)

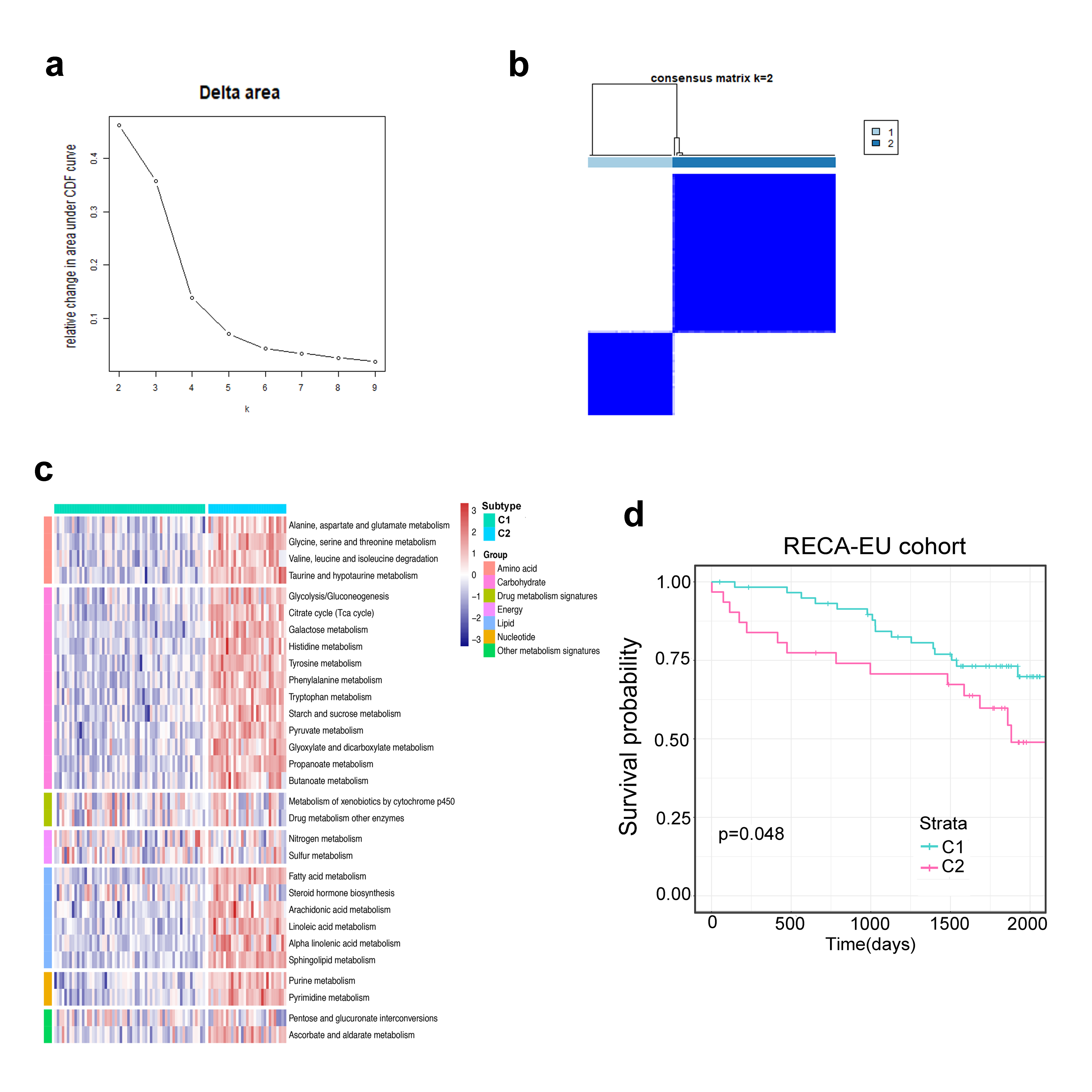

Supplement: Supplementary Figure 1 — Two distinct metabolism subtypes are validated in RECA-EU cohort. (a, b) The CDF curves of consensus matrix indicates that when k=2, the interference between subgroups is minimal. (c) Heatmap of the seven categories of metabolism pathways for two subtypes (C1 and C2).(d) Survival analysis between two metabolism subtypes. P value is given by log rank test. [file Image_1.tif]

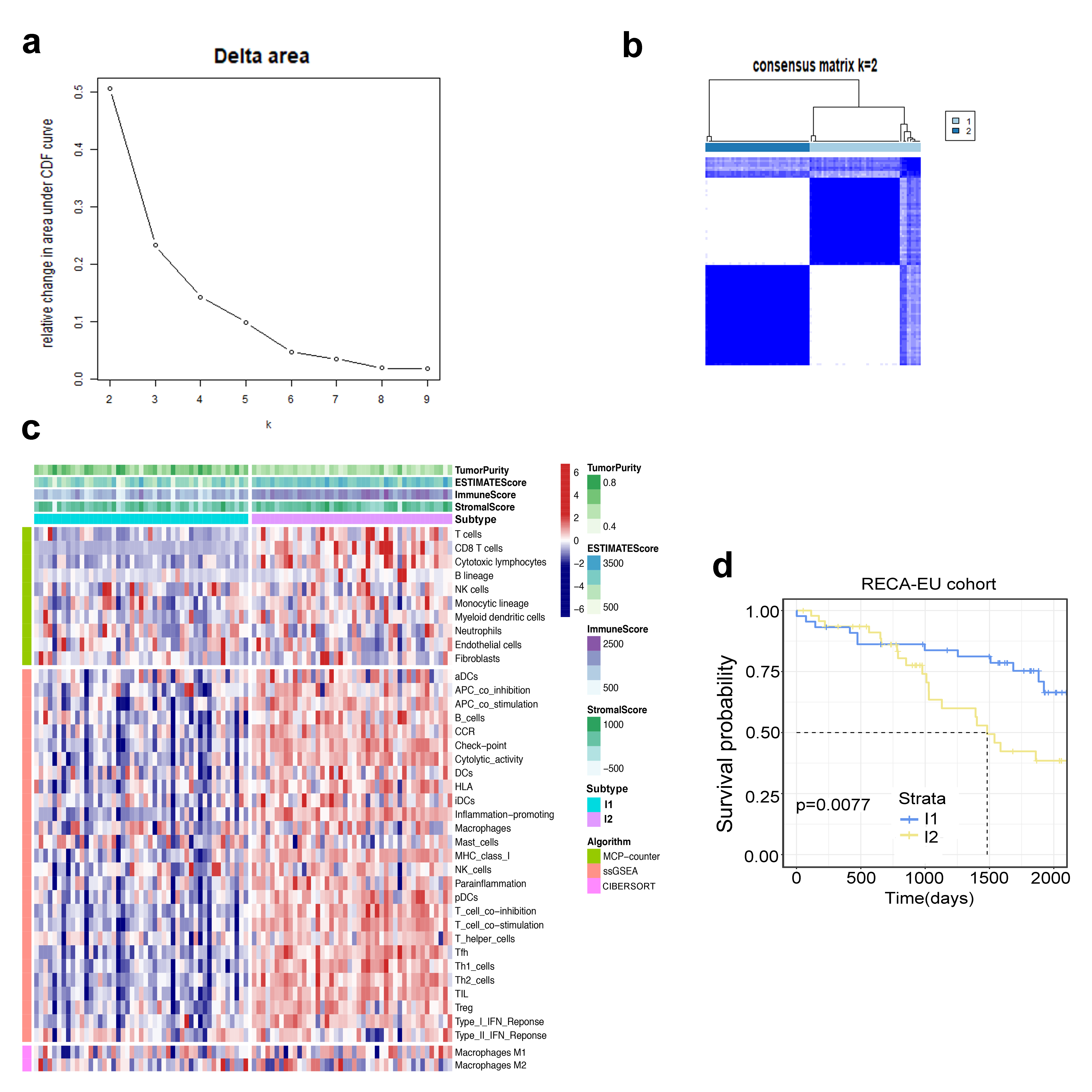

Supplement: Supplementary Figure 2 — Two distinct immune subtypes are validated in RECA-EU cohort. (a, b) The CDF curves of consensus matrix indicates that when k=2, the interference between subgroups is minimal. (c) Heatmap of the aboundance of immune components infiltration for two subtypes (I1 and I2). (d) Survival analysis between two immune subtypes. P value is given by log rank test. [file Image_2.tif]

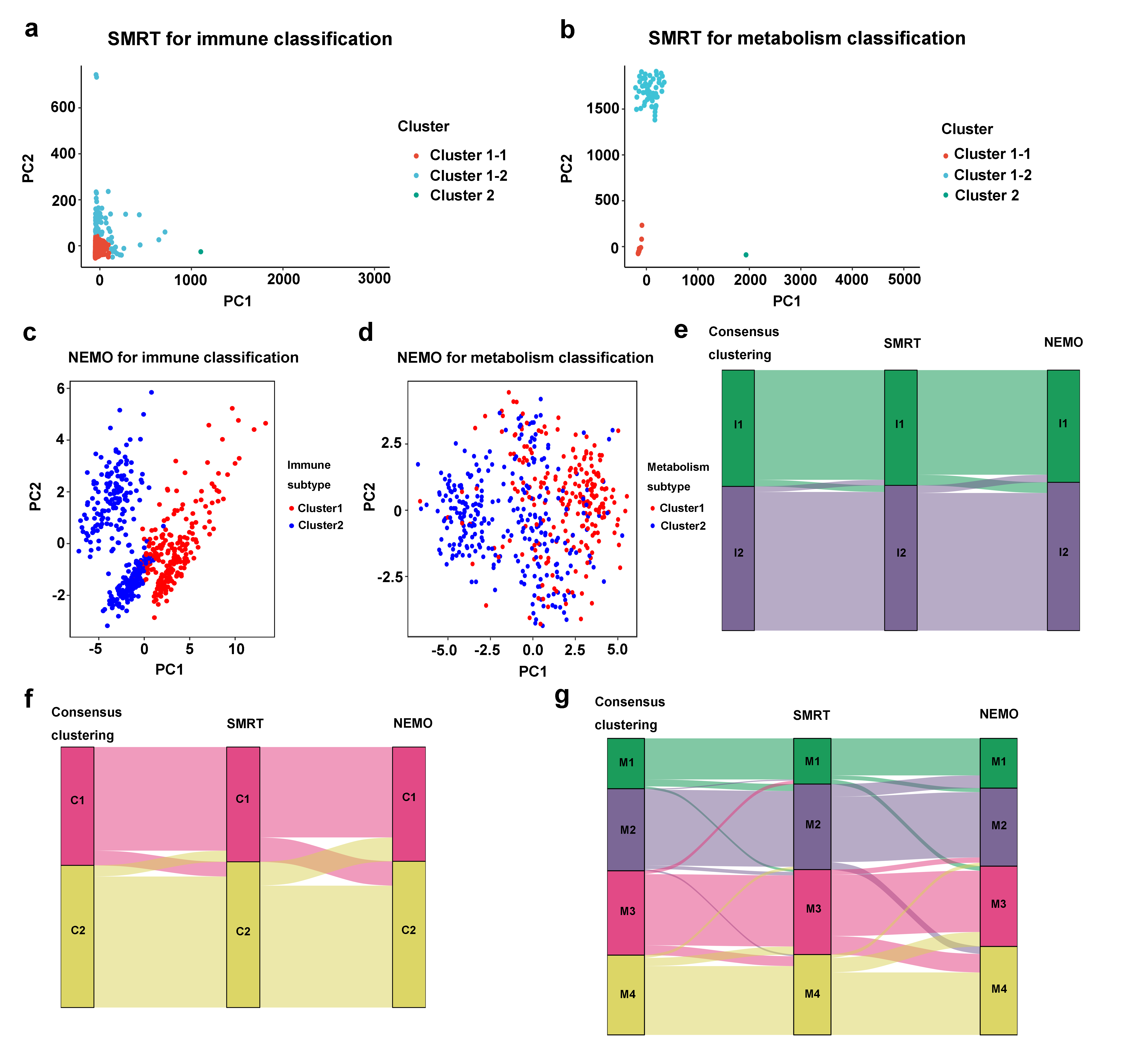

Supplement: Supplementary Figure 3 — Immune and metabolism classification results in TCGA-KIRC cohort generated by SMRT and NEMO clustering method show good consistency with consensus clustering. (a, b) PCA plots show that samples can be mainly classified into two immune and metabolism subtypes by SMRT clustering method. (c, d) PCA plots show that two immune and metabolism subtypes can be separated clearly using NEMO clustering method. (e-g) Sankey diagram shows that the three clustering methods, consensus clustering, SMRT and NEMO have significant overlapping samples in immune subtypes (e), metabolism subtypes (f) and immunometabolism subtypes (g). [file Image_3.tif]

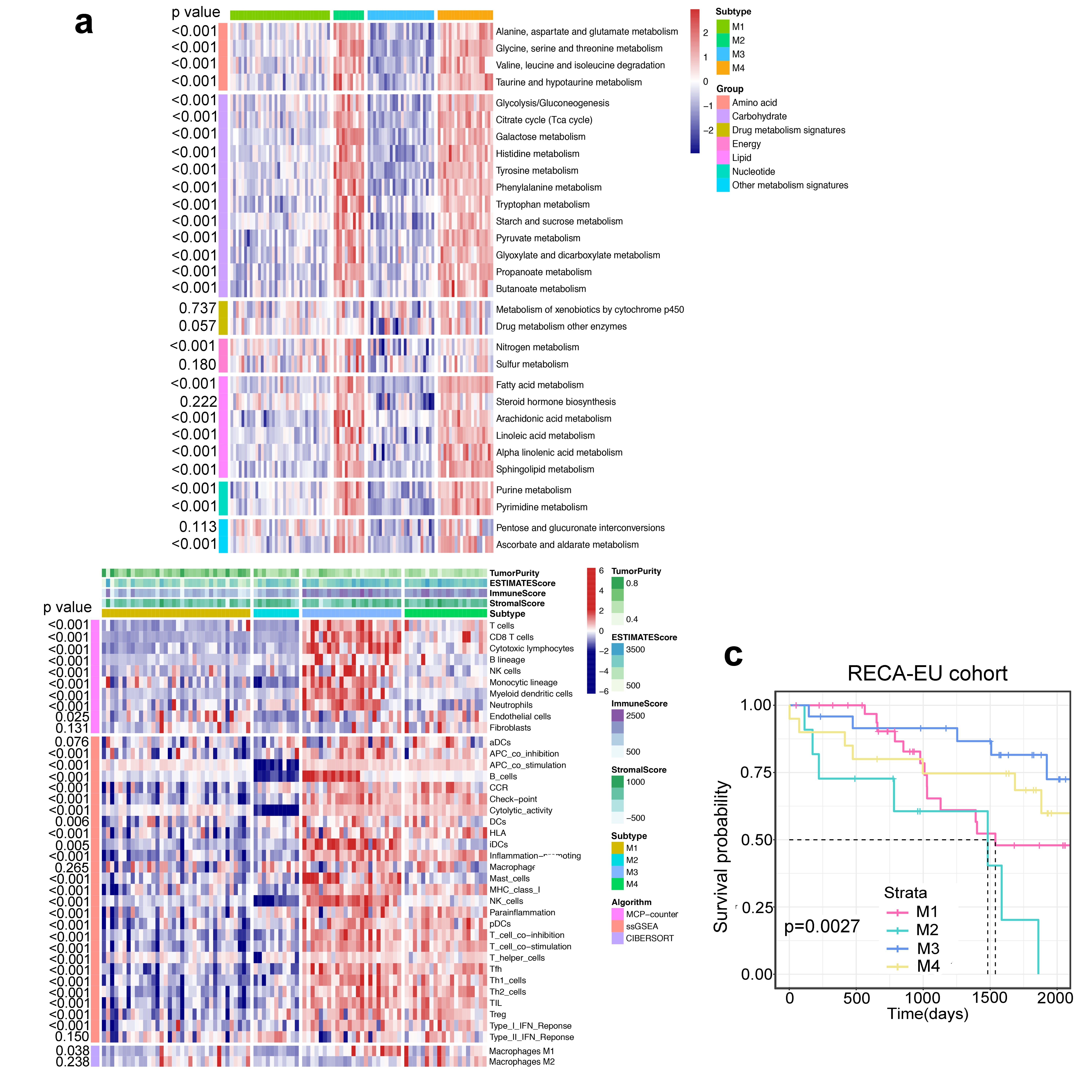

Supplement: Supplementary Figure 4 — Validation of four immunometabolism subtypes in RECA-EU cohort. (a, b) Heatmaps of metabolism and immune related signatures in four immunometabolism subtypes (M1, M2, M3 and M4). P value is calculated by ANOVA test. (c) Comparison of survival outcomes among four subtypes. P value is given by log rank test. [file Image_4.tif]

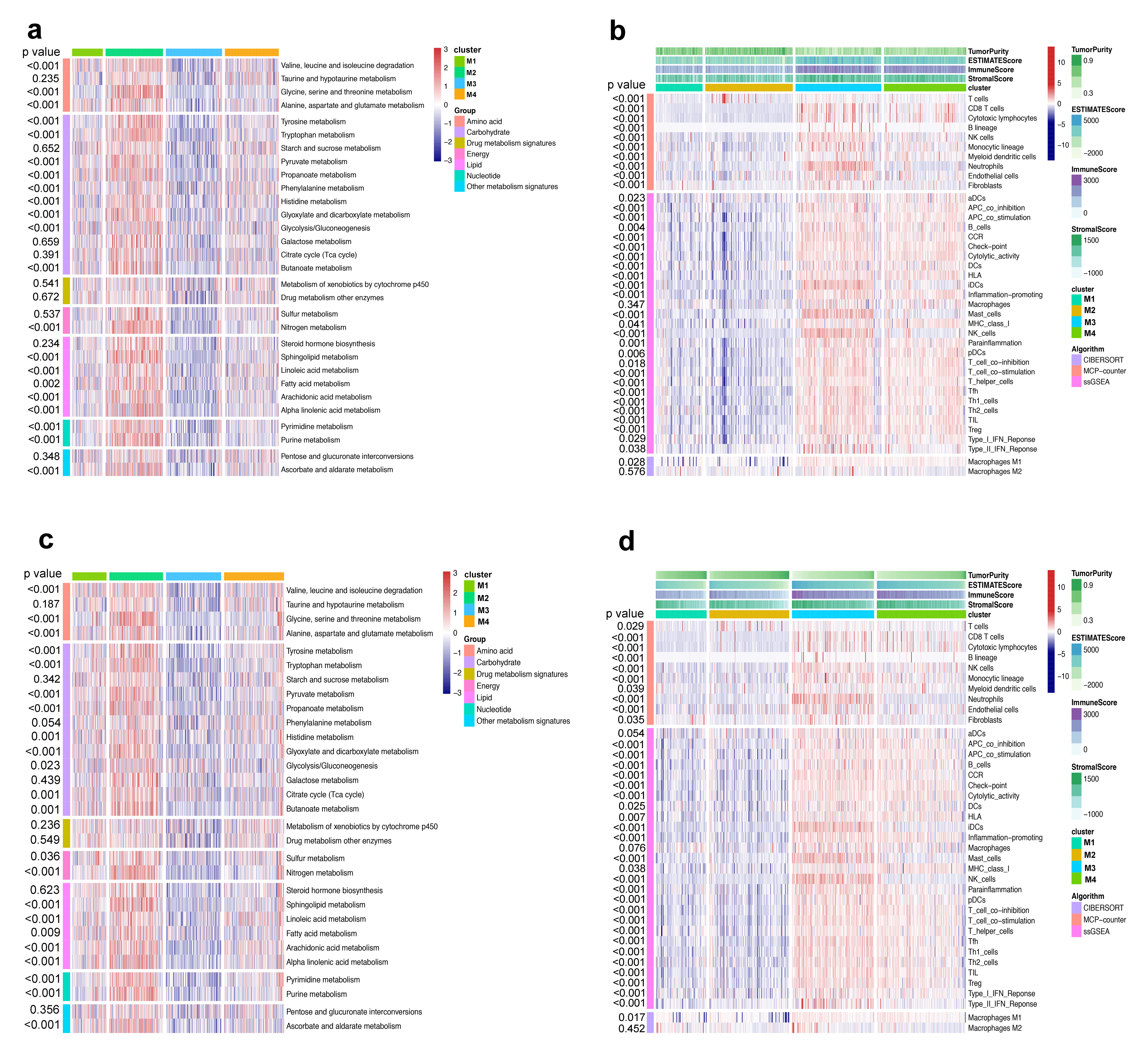

Supplement: Supplementary Figure 5 — The immune and metabolism landscapes of the four immunometabolism clusters in TCGA-KIRC cohort generated by SMRT and NEMO clustering methods. (a, b) Heatmaps of metabolism and immune related signatures in four immunometabolism subtypes (M1, M2, M3 and M4) generated by SMRT clustering method. P value is calculated by ANOVA test. (c, d) Heatmaps of metabolism and immune related signatures in four immunometabolism subtypes (M1, M2, M3 and M4) generated by NEMO clustering method. P value is calculated by ANOVA test. [file Image_5.tif]

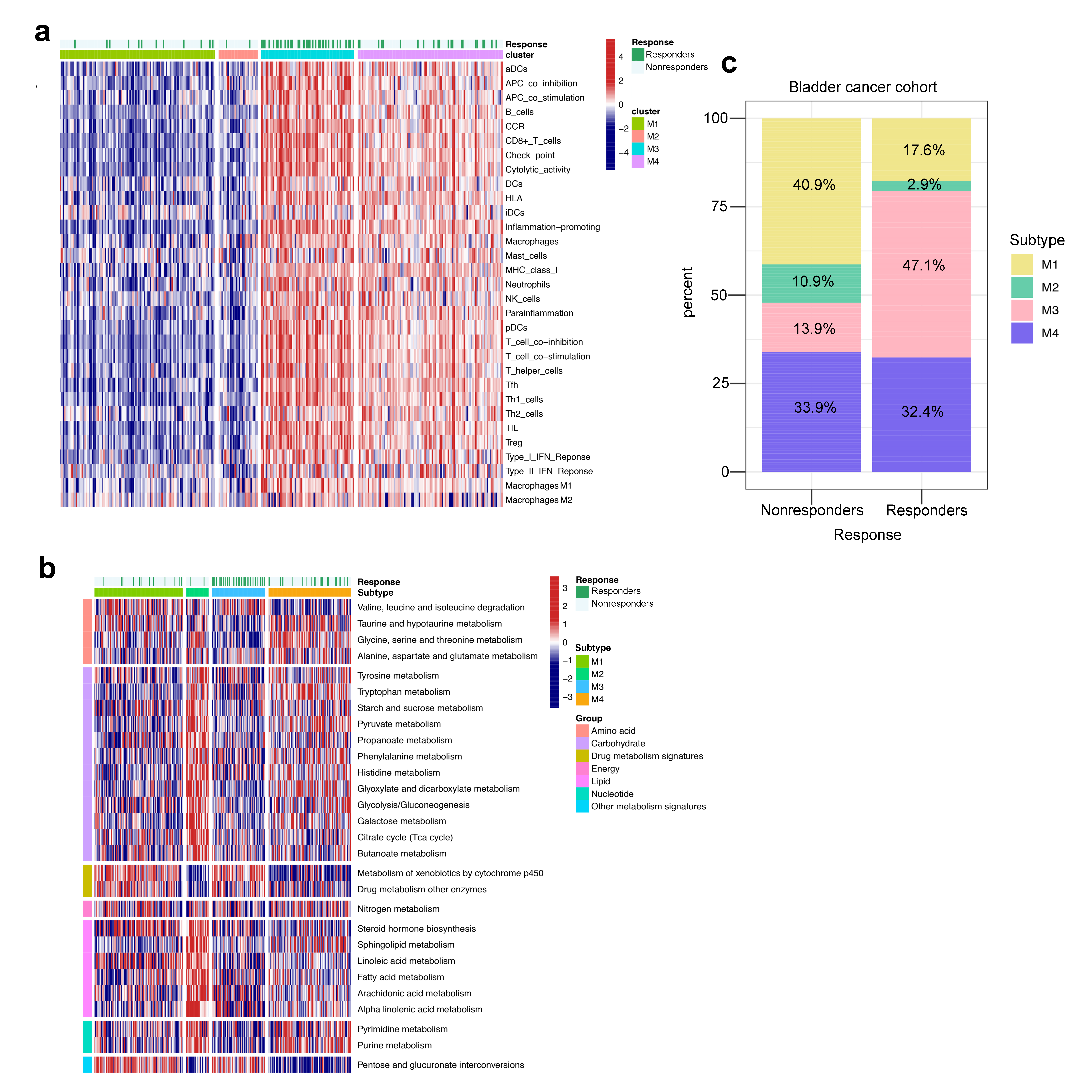

Supplement: Supplementary Figure 6 — Immunometabolism subtypes of bladder cancer correlate with immunotherapy. (a, b) Heatmap shows the immune landscape and metabolism signatures of four immunometabolism subtypes in bladder cancer prior to anti-PD-1 therapy (IMvigor210 cohort). (c) Percentages of four immunometabolism subtypes in responders and nonresponders prior to anti-PD-1 therapy in bladder cancer. [file Image_6.tif]
